# Dried Beetroots: Optimization of the Osmotic Dehydration Process and Storage Stability

**DOI:** 10.3390/foods13101494

**Published:** 2024-05-11

**Authors:** Danijela Šuput, Slađana Rakita, Nedeljka Spasevski, Ružica Tomičić, Danka Dragojlović, Senka Popović, Nevena Hromiš

**Affiliations:** 1 Faculty of Technology Novi Sad, University of Novi Sad, Bulevar cara Lazara 1, 21000 Novi Sad, Serbia; ruzica.tomicic@uns.ac.rs (R.T.); madjarev@uns.ac.rs (S.P.); nevena.krkic@uns.ac.rs (N.H.); 2 Institute of Food Technology, University of Novi Sad, Bulevar cara Lazara 1, 21000 Novi Sad, Serbia; sladjana.rakita@fins.uns.ac.rs (S.R.); nedeljka.spasevski@fins.uns.ac.rs (N.S.); danka.dragojlovic@fins.uns.ac.rs (D.D.)

**Keywords:** beetroot, drying, sugar beet molasses, storage, edible coating, quality

## Abstract

In this study, beetroots were osmotically dehydrated in sugar beet molasses. The input parameters of the drying process were varied: temperature (20 °C, 40 °C, and 60 °C), time (1 h, 3 h, and 5 h), and concentration of sugar beet molasses (40%, 60%, and 80%). Basic quality indicators were determined for the dried beetroot samples: dry matter content, water loss, solid gain, mineral and betaine content, and phenols and flavonoids, as well as antioxidant potential. After optimizing the results, favorable drying parameters were selected: temperature 60 °C, molasses concentration 70%, and processing time 5 h. According to the optimal drying conditions, the beetroots were dried and stored at 4 °C for 28 days. Half of the dried samples were coated with an edible biopolymer coating based on *Camelina sativa* oilcake, while the other half of the samples remained uncoated. The sustainability study aimed to confirm the effects of the biopolymer coating on the quality and sustainability of the osmotically dried beetroots.

## 1. Introduction

The *Beta vulgaris*, also referred to as red beet, table beet, garden beet, or simply beet, is a type of root vegetable belonging to the *Chenopodiaceae*, a subfamily of the *Amaranthaceae* family of flowering plants [1]. The taste, appetite stimulation, ease of digestion, and refreshing character of beetroot make it an appealing and widely grown crop [2]. Its contents are 87.58% water, 9.56–9.96% carbohydrates, 2–2.8% fiber, 1.61–1.68% protein, and 0.17–0.18% fat [3]. Sucrose is the main sugar found in beetroots [4]. In addition, beetroots are an excellent source of micronutrients, vitamins, minerals, and both essential and non-essential amino acids [3]. Betalains, approved by the European Union as E-162, give red beets their distinctive hue and nowadays are widely used as natural food coloring agents [5]. Betalains are water-soluble, nitrogenous, reddish-violet or yellow-orange hydrophilic chemicals that are typically found in roots, fruits, stems, and flowers [6]. Two groups of chemicals are known as betalains: betaxanthin (yellow-orange) and betacyanin (reddish-violet). Up to 90% of the red pigment in beetroots is made up mostly of betain, the major betacyanin [3]. Approximately 200 mg of this pigment is found in every 100 g of red beetroot, but light, heat, and oxygen easily cause it to degrade [7]. Furthermore, betalains account for 70–100% of beetroot’s total phenolics [5], and as these are known for their antimicrobial, antioxidant, and anti-inflammatory qualities. They can be used as natural food preservatives as well as to create functional foods that have positive health effects [6]. Beetroots contain smaller levels of flavonoids and hydroxycinnamic acids, including gallic, syringic, and caffeic acids, in addition to betalains [8]. Different culinary traditions employ beetroots in various processed forms, such as powder, bread, gel, cooked, oven-dried, pickled, pureed, or jam [9]. Growing new, promising beetroot varieties is important, but so is maintaining their quality during storage to generate high-quality processed products [10]. Plant commodities frequently spoil, owing to a variety of circumstances, which results in substantial waste [11]. Beetroots can be dried to prevent waste, and thereby capitalize on agricultural surpluses, and add value in the off-season. Drying not only offers an alternate way to lessen post-harvest damage, but it also lengthens shelf life, decreases weight and volume, and makes storage and transportation easier [12]. According to Adil et al. [13], traditional drying techniques used in food processing to increase shelf life may cause physical, chemical, and sensory changes in the final product. Nowadays, numerous food preservation techniques are applied to maintain the desired quality while extending sustainability: freeze-drying [14], pulsed electric fields [15], microwave fluidization technology [16], light irradiation [17], ultrasound [18], etc.

In recent years, foods with an intermediate moisture content (IMFs) have gained popularity compared to completely dried food. Fruits and vegetables with intermediate moisture offer advantages over typically dried ones. The product is achieved by removing or binding only a small amount of water, often by adding a humectant, to slow down microbial growth, as opposed to removing the majority of the water [19]. Despite being one of the oldest preservation methods, dehydration is still frequently applied in the food sector. Since osmotic dehydration (OD) can be used to produce intermediate-moisture-content (semi)products, such as dried fruits and vegetables, it can be considered as both a pretreatment for drying and a processing method [20]. It is based on the decrease in water activity (a_w_) and involves processes of degradation, especially those induced by microbial and chemical agents. As a result, this process modifies the product’s texture, color, and volume, as well as lowers its weight and volume, making storage and transportation easier [21]. By immersion in aqueous solutions with high osmotic pressure, a process known as osmotic dehydration can remove water while increasing the number of soluble solids [22]. Osmotic agents come in a variety of forms, including sodium chloride, glucose, and corn syrup [23]. During osmotic dehydration, mass transfer occurs in two directions: solute diffuses from the solution into the biomaterial and water diffuses from the biomaterial to a hypertonic solution. In osmotically dehydrated biomaterial, the rate of solid gain (SG) is typically lower than the rate of water loss (WL) [24]. The osmotic pressure of the hypertonic solution is what propels the water transport from the biomaterial tissue into the solution [25]. According to Kowalski and Łechtańska [7], the dehydration process continues until the chemical potentials between the biomaterial cell and the osmo-active fluid are balanced. By optimizing the osmotic dehydration process of beetroot in sugar solution Singh and Hathan [22] highlighted the sample geometry, solution agitation, solution/product mass ratio, process duration, osmotic medium concentration, size and geometry of the material, osmotic medium variations, maturity level, and physical and chemical characteristics of the initial product as factors influencing OD efficiency. Osmotic dehydration enhances food’s sensory and functional qualities.

Although osmotic dehydration is a traditional method, and although beetroots have been dried and characterized in various ways so far, the current literature shows that beetroots have not been osmotically dehydrated in sugar beet molasses solution yet. This work aims to optimize the dehydration conditions, and also to monitor the sustainability of the obtained semi-product. This work set out to optimize the osmotic dehydration process of beetroots in sugar beet molasses, focusing on the choices of optimal process duration, working temperature, and concentration of the osmotic solution. Determining these parameters was necessary in order to obtain intermediate-moisture-content beetroots with the highest-ranked physico-chemical and nutritional properties. In the second phase of the work, the optimal parameters were applied to the beetroots, and the changes in the quality of the osmotically dehydrated beetroots as a semi-finished product were evaluated to determine the direction of further application. As an adjunct to the work, a biopolymer coating based on wild flax (*Camelina sativa*) was used to determine whether it would have a positive effect on the sustainability of the dehydrated beetroots. The obtained semi-product (OD beets) could be considered a functional food with an enriched nutritional composition. The final purpose is to implement it as a component in raw bars, vegan food, salads, bakery products, and healthy snacks.

## 2. Materials and Methods

### 2.1. Materials

Beetroots (Kestrel Sakata hybrid, moisture content 88.05%) used in this study were purchased from a local grower prior to testing, peeled, and cut into 1 cm^3^ cubes using a sterile knife, after which they were subjected to the osmotic treatment in sugar beet molasses.

Sugar beet molasses (dry matter content 85%) was supplied by the sugar factory “Crvenka” (Crvenka, Serbia).

The fraction of cold-pressed *Camelina sativa* seed cake finer than 180 μm (CSoC) was kindly provided by the Institute of Food Technology and it served for biopolymer coating preparation.

All other reagents used in this study were of analytical grade.

### 2.2. Experimental Design—Chemometric Analysis

The experimental data used for the optimization study were obtained using a Box and Behnken experimental design (3 level–3 parameters) with 15 runs. The independent variables were temperatures (*T*) of 20, 40, and 60 °C; time (*t*) of 1, 3, and 5 h; and molasses concentrations (*Conc*) of 60, 70, and 80%. Based on the data from 15 runs, we opted for the sample that had the best properties regarding the quality parameters, and in the second part of the experiment, its storage stability (coated with biopolymer film and without biofilm) was monitored in terms of quality and safety parameters.

### 2.3. Osmotic Dehydration Parameters

Based on the following expressions, the responses of the osmotic dehydration process were calculated.

Dry matter content (*DMC*),

(1) $$DMC=\frac{m_{d}}{m_{i}}\times 100\%$$

Water loss (*WL*),

(2) $$WL=\frac{m_{i}\cdot z_{i}-m_{f}\cdot z_{f}}{m_{i}}\times [g/g_{f.s.}]$$

Solid gain (*SG*) [26],

(3) $$SG=\frac{m_{f}\cdot s_{f}-m_{i}\cdot s_{i}}{m_{i}}\times [g/g_{f.s.}]$$

For all three equations,

*m*_*d*_—a mass of dry matter [g];
*m*_*i*_—a mass of fresh sample [g_f.s._];
*m*_*f*_—a mass of sample after the osmotic dehydration process [g];
*z*_*i*_—a mass fraction of water in the fresh sample [g/g_f.s._];
*z*_*f*_—a mass fraction of water in the sample after the osmotic dehydration [g/g];
*s*_*i*_—a mass fraction of dry matter in the fresh sample [g/g_f.s._];
*s*_*f*_—a mass fraction of dry matter in the sample after the osmotic dehydration [g/g];
_f.s._—fresh sample.

Dry matter content in fresh and osmotically dehydrated beetroot samples was determined gravimetrically by drying at 105 °C in an oven (Instrumentaria, Zagreb, Croatia) until a constant weight was reached.

### 2.4. Methods for Assessing Beetroot Quality

#### 2.4.1. Sample Preparation

The samples of beetroot were crushed in a laboratory mill and cooled (KN 295 Knifetec, FOSS, Hillerød, Denmark) before analyses of titratable acidity, betaine, and mineral content. In order to prepare extracts for the determinations of total phenolic and flavonoid contents, as well as for DPPH and ABTS quenching activity tests, the ground samples (5 g) were homogenized with 30 mL of methanol/water solution (80/20, *v*/*v*) and kept at 25 °C in an ultrasound bath (T1-H-1S, Elma Schmidbauer GmbH, Singen, Germany) for 15 min. A suspension was further filtered through a filter paper (grade: 1288; Munktell & Filtrak GmbH, Bärenstein, Germany), and the obtained extract was used for the analysis. Three replications for each sample were conducted for methods in Section 2.4.1, Section 2.4.2, Section 2.4.3, Section 2.4.4, Section 2.4.5 and Section 2.4.6.

#### 2.4.2. Titratable Acidity

Acidity was determined according to the Rulebook on methods of taking samples and performing chemical and physical analyses for quality control of fruit and vegetable products (Official Gazette of the SFRJ, No. 29/83) [27].

#### 2.4.3. Antioxidative Activity

The DPPH radical scavenging activity was determined in the manner suggested by Peinado et al. [28] and Bajić et al. [29]. A solution of 1 mM DPPH in methanol was prepared 24 h before analysis and kept overnight at room temperature in the dark. For the analysis, 100 μL of the sample extract was mixed with 3.9 mL of DPPH reaction solution and incubated for 1 h in the dark. The range of investigated concentrations was 0.04–0.04 mg/mL. The decrease of absorbance was measured at 517 nm. The results were expressed as IC_50_ (mg/mL) (inhibitory concentration), which is the concentration of sample extract required for 50% scavenging of DPPH radicals.

The ABTS radical cation test was performed based on the method described by Re et al. [30]. ABTS radical cation stock solution was prepared by dissolving ABTS in 0.1 M phosphate buffer (pH 7.40) containing 5 mM NaCl (PBS). ABTS radical cation was produced by reacting ABTS solution with 2.45 mM potassium persulfate (final concentration). The solution was prepared a day before analysis, and the mixture left to stand in the dark to enable the production of radicals. Then, the ABTS radical cation solution was diluted with PBS to an absorbance value of 0.7 at 734 nm. The range of investigated concentrations was 0.05–0.05 mg/mL. An aliquot of the sample extract (30 μL) was mixed with 3 mL ABTS radical cation solution and absorbance was measured after 10 min. The results were expressed as IC_50_ (mg/mL), which is the concentration of sample extract required to inhibit 50% of the available ABTS radical free radicals.

#### 2.4.4. Total Flavonoid Content

The total flavonoid content test was performed according to the methods described in Kim et al. [31]. For this analysis, a 100 μL aliquot of the sample extract was mixed with 4 mL of distilled water in a test tube. Subsequently, 0.3 mL of 5% NaNO_2_ was added, and after a 5 min interval, 0.3 mL of 10% AlCl3 was introduced, ensuring a thorough mixing. After incubation at room temperature for 6 min, 2 mL of 1 M NaOH and 2.4 mL of distilled water were added to the mixture. The absorbance was then measured at 415 nm. Results were expressed as milligrams of catechin equivalents (ECA) per 100 g of dry matter (mg ECA/100 g d.m.).

#### 2.4.5. Total Phenolic Content

Determination of total phenolic content was carried out based on the Folin–Ciocalteu reaction [32], using gallic acid as a standard. Briefly, 100 μL of sample extract was diluted with 7.9 mL of distilled water and mixed with 0.5 mL of 0.67 N Folin–Ciocalteu reagent (PanReac AppliChem, Darmstadt, Germany). The mixture was allowed to stand at room temperature for 5 min before adding 1.5 mL of 20% Na_2_CO_3_. After incubation at room temperature in the dark for 1 h, the absorbance was measured at 760 nm using a UV-Vis spectrophotometer (Jenway 6300, Cole-Parmer, Vernon Hills, IL, USA). Total phenolic content was expressed as milligrams of gallic acid equivalents per 100 g of dry matter (mg eq GA/100 g d.m.).

#### 2.4.6. Betaine Content

The beetroot samples were prepared according to the method described in Kojić et al. [33] to determine betaine content. In brief, the ground sample (0.5 g) was suspended in 25 mL of methanol and vortexed for 10 min. Following a 30 min ultrasonic extraction in an ultrasound bath at room temperature, the sample underwent vigorous shaking and was then centrifuged for 10 min at 5000 rpm (Eppendorf Centrifuge 5804R, Eppendorf, Wien, Austria). The resulting upper methanol layer was evaporated to dryness. Then, the residue was reconstituted in 2 mL of water and filtered through a membrane filter based on regenerated cellulose (pore size 0.22 mm, diameter 25 mm) to obtain the sample extract. An HPLC system (Agilent Technologies Inc., Santa Clara, CA, USA) fitted with an ELSD detector (1290 Infinity ELSD, Agilent Technologies Inc., Santa Clara, CA, USA) and a Kinetex HILIC (Phenomenex, Aschaffenburg, Germany) column (2.6 µm, 100 × 2.1 mm) was used to quantify the betaine content. The mobile phase for the analysis was an 80:20 combination of acetonitrile and 10 mM acetate buffer (pH 3.7), with a flow rate of 0.5 mL/min. Ten minutes was the whole run time, and there was a 5 µL injection volume.

#### 2.4.7. Mineral Content

Mineral content was determined according to ISO Standard 6869 [34], which is based on atomic absorption spectrometry, using a flame atomic absorption spectrometer (Varian SPECTRA AA-10, Varian Techtron Pty Limited, Mulgrave, Victoria, Australia) equipped with a flame furnace and operated with an air acetylene flame, after mineralization by dry aching.

#### 2.4.8. Microbiological Analysis

To assess the microbiological quality of osmotically dehydrated beets, as well as osmotically dehydrated beets coated with *Camelina sativa* film stored in the fridge at 7 °C after 7, 14, 21, and 28 days, microbiological determinations were carried out according to the International Organization for Standardization (ISO) methods. Namely, determination of the *Escherichia coli* was accomplished by ISO 16649-2 [35], determination of *Enterobacteriaceae* was accomplished by ISO 21528-1 [36], determination of *Salmonella* spp. was accomplished by ISO 6579 [37], determination of *Listeria monocytogenes* was accomplished by ISO 11290-2 [38], determination of *Clostridium perfringens* was accomplished by ISO 7937 [39], and the total number of microorganisms was determined in accordance with ISO 4833-1 [40], while the total number of yeasts and molds was determined by using the method described in ISO 21527-1 [41].

Briefly, a mass of 25 g of each sample was solubilized in 225 mL of sterile buffered peptone water (HiMedia Laboratories, Mumbai, India) in a stomacher bag and homogenized (Homogeniser, easyMIX, VWR) for 2 min before dilution was made. Next, ten-fold serial decimal dilutions were performed for each sample, and aliquots were transferred to culture media specific for each microbial group. Plating was performed in duplicate, and results were expressed in colony-forming units per gram (CFU/g).

Subsequently, to enumerate the beta-glucuronidase-positive *E. coli*, 1 mL of diluted sample was dispersed in Tryptone Bile X-Glucuronide Medium (TBX, HiMedia Laboratories, India), and plates were incubated at 44 °C for 24 h. In the case of *Enterobacteriaceae*, culture media composed of Violet Red Bile Glucose Agar (VRBG, HiMedia Laboratories, India) were incubated at 37 °C for 24 h. *Salmonella* was detected and enumerated as recommended in the standard protocol. Therefore, 0.1 mL and 1 mL of the aliquot were transferred to 10 mL of Rappaport Vassiladis Soyabean Meal Broth (RVSM broth, HiMedia Laboratories, India) and 10 mL of Muller Kauffmann Tetrathionate Novobiocin Broth (MKTT broth, HiMedia Laboratories, India), respectively, and incubated overnight at 42 °C and 37 °C, respectively. Then, the enriched solution of RVSM and MKTT was streaked on Xylose-Lysine-Deoxycholate Agar (XLD, HiMedia Laboratories, India) and incubated at 37 °C for 24 h. For the enumeration of *L. monocytogenes*, 0.1 mL of diluted sample was spread on Listeria Ottaviani Agosti Agar (ALOA, HiMedia Laboratories, India) and incubated overnight at 37 °C. Further, the count of *Clostridium perfringens* was performed by the pour-plating method on Tryptose Sulfite Cycloserine Agar (TSC, HiMedia Laboratories, India) after incubation under anaerobic conditions at 37 °C for 24 h. Finally, the total count of microorganisms was performed on standard Plate Count Agar (PCA, HiMedia Laboratories, India) incubated at 30 °C for 72 h, while the total number of yeasts and molds was determined using Dichloran Rose Bengal Chloramphenicol Agar (DRBC, HiMedia Laboratories, India) after incubation at 25 °C for 5 days.

### 2.5. Storage Stability of Osmotically Dehydrated Beetroots

Osmotically dehydrated beetroot samples were stored at (4 ± 0.5) °C and sampled after 7, 14, 21, and 28 days for the analyses. One-half of the samples were unchanged and used as control, while the other half of the samples were coated with *Camelina sativa* (CSoC)-based biopolymer coating. The coating suspension was prepared as described by Šuput et al. [42]. In brief, 5% CSoC was dispersed in distilled water with 40% glycerol (*w*/*w*) as a plasticizer. The suspension pH was adjusted to pH 10, incubated at 100 °C in a waterbath for 20 min, and finally filtrated. Osmotically dehydrated beetroot samples were immersed in coating suspension for 30 s and left for 10 min to drain. The dipping treatment was repeated 3 times. Samples without biopolymer coating were labeled as “OD”, while samples with biopolymer coating were labeled as “ODC”, and both were analyzed after 7, 14, 21, and 28 days. The following quality parameters were monitored: acidity, antioxidative activity change by DPPH and ABTS tests, flavonoids, phenols, and betaine content, as well as microbiological profile.

### 2.6. Statistical Analysis

#### 2.6.1. Principal Component Analysis (PCA)

PCA was utilized to uncover relationships among variables demonstrating similar interactions. The data resulting from the PCA analysis of 15 beetroot samples were visually depicted through biplots. The analysis was conducted using StatSoft Statistica 12 software developed by StatSoft Inc., based in Tulsa, OK, USA.

#### 2.6.2. Standard Scores

The ranking of 15 samples was performed by comparing their raw data to the extreme values, following the method by Brlek et al. [43]. Criteria for ranking included parameters like *DMC*, *WL*, *SG*, Mg, K, Na, Ca, *DPPH*, *ABTS*, flavonoid and phenol content, acidity, and betaine content.

#### 2.6.3. Artificial Neural Network (ANN) Model

The dataset for ANN modeling, consisting of 15 samples, as detailed earlier, was divided into training (60%), cross-validation (20%), and testing (20%) sets. To enhance accuracy, both input and output standardization were implemented through min–max normalization. The suggested multilayer perceptron model (MLP) was structured with three layers, featuring a feedforward architecture and employing backpropagation for training [29]. The hidden layer contained 5 to 10 neurons, and various activation functions (tangent, sigmoidal, exponential, and identity) were tested. The Broyden–Fletcher–Goldfarb–Shanno (BFGS) algorithm was used to build the ANN model, and iteratively adjusted weights and biases using 100,000 different configurations. The objective was to minimize square error until both learning and cross-validation curves approached zero. The coefficients for the hidden layer were represented by matrices *W*_1_ and *B*_1_, while the coefficients for the output layer were represented by matrices *W*_2_ and *B*_2_:

(4) $$Y=f_{1}\left(W_{2}\cdot f_{2}\left(W_{1}\cdot X\right)+B_{1}\right)+B_{2}$$

where *f*_1_ and *f*_2_ are transfer functions in the hidden and output layers, respectively, and *X* is the input vector.

#### 2.6.4. Sensitivity Analysis

Yoon’s interpretation method was employed to assess the impacts of *t*, *T*, and *conc* on *DMC*, *WL*, *SG*, Mg, K, Na, Ca, *DPPH*, *ABTS*, flavonoid and phenol content, acidity, and betaine content. This assessment utilized a weight coefficient derived from the constructed ANN model.

(5) $$RI_{ij}\left(\%\right)=\frac{\sum_{k=0}^{n}\left(w_{ik}\cdot w_{kj}\right)}{\sum_{i=0}^{m}\left|\sum_{k=0}^{n}\left(w_{ik}\cdot w_{kj}\right)\right|}\cdot 100\%$$

where *w*—weight coefficient in ANN model, *i*—input variable, *j*—output variable, *k*—hidden neuron, *n*—number of hidden neurons, and *m*—number of inputs.

The model’s accuracy was evaluated through various standard computational tests, including the coefficient of determination (*r*^2^), reduced chi-square (*χ*^2^), mean bias error (*MBE*), root mean square error (*RMSE*), mean percentage error (*MPE*), the sum of squared errors (*SSE*) and average absolute relative deviation (*AARD*).

(6) $$\chi^{2}=\frac{\sum_{i=1}^{N}{\left(x_{exp,i}-x_{pre,i}\right)}^{2}}{N-n}$$

(7) $$RMSE={\left[\frac{1}{N}\cdot \overset{N}{\underset{i=1}{\sum }}{\left(x_{exp,i}-x_{pre,i}\right)}^{2}\right]}^{1/2}$$

(8) $$MBE=\frac{1}{N}\cdot \overset{N}{\underset{i=1}{\sum }}\left(x_{exp,i}-x_{pre,i}\right)$$

(9) $$MPE=\frac{100}{N}\cdot \overset{N}{\underset{i=1}{\sum }}\left(\frac{\left|x_{exp,i}-x_{pre,i}\right|}{x_{pre,i}}\right)$$

(10) $$SSE=\overset{N}{\underset{i=1}{\sum }}{\left(x_{exp,i}-x_{pre,i}\right)}^{2}$$

(11) $$AARD=\frac{100}{N}\cdot \overset{N}{\underset{i=1}{\sum }}\left|\frac{x_{exp,i}-x_{pre,i}}{x_{pre,i}}\right|$$

(12) $$r^{2}=1-\frac{\sum_{i=1}^{N}{\left(x_{exp,i}-x_{pre,i}\right)}^{2}}{\sum_{i=1}^{N}{\left(x_{exp,i}-\bar{x}\right)}^{2}},\bar{x}=\overset{N}{\underset{i=1}{\sum }}x_{exp,i}$$

where *N* represents the total number of data records, while *x_exp_*_,*i*_ and *x_pre_*_,*i*_ are the experimental and model predicted values, respectively.

## 3. Results and Discussion

### 3.1. OD Parameters—DMC, WL, and SG

After analyzing all 15 runs, *DMC* was within a wide range, from 16.574% to 50.149% (Table 1). *DMC* obtained at a temperature of 20 °C was in the range of 16.574–36.969%; at the temperature of 40 °C, it was 22.921–47.999%; and at the temperature of 60 °C, it was 26.790–50.149%. *DMC* obtained with the application of molasses solution with a concentration of 60% was in the range of 22.921–38.287%, that obtained with a concentration of 70% was in the range of 16.574–50.149%, and that obtained with a concentration of 80% was in the range of 23.273–47.999%. *DMC* obtained after 1 h of osmotic dehydration was in the range of 16.574–26.790%, that obtained after 3 h was in the range of 26.737–43.583%, and that obtained after 5 h was in the range of 36.969–50.149%. Higher values of each applied process parameter led to a higher content of dry matter in beetroots after the osmotic dehydration procedure.

Water loss and solid gain were different for different molasses temperatures, concentrations, and process durations (Table 1). The minimal water loss was 0.333 g/g_f.s._ (run 5), corresponding to low processing inputs (20 °C, 70%, 1 h), while the maximal water loss was 0.710 g/g_f.s._ (run 12), corresponding to high processing outputs (40 °C, 80%, 5 h). Low solid gain values (lower than 0.015 g/g_f.s._) were recorded for runs 1, 3, 5, 9, 10, 13, and 15. A shared characteristic of these runs is that they were not performed at maximal temperature or maximal time. Furthermore, the maximum concentration of molasses did not contribute to higher values of solid gain. On the other hand, the maximal solid gain was associated with run 8 (0.050 g/g_f.s._), which was performed at high input parameters: maximal temperature (60 °C), medium molasses concentration (70%), and maximal process duration (5 h).

It can be concluded that the efficiency of the osmotic dehydration procedure was more successful, and therefore the *DM*, *WL*, and *SG* values were higher, at higher values of the input process parameters. Low temperature–low concentration–low time conditions caused lower *DM*, *WL*, and *SG* values, while high temperature–high concentration–high time conditions provided higher *DM*, *WL*, and *SG* values. The obtained results are in accord with Nićetin et al. [44], who osmotically dehydrated celery root in sugar beet molasses and ternary solution of water, sucrose, and salt; with Prajapat et al. [19], who osmotically dehydrated beetroot tutti-frutti in sugar syrup; and with Soquetta et al. [45], who investigated the effect of an ultrasonic bath in osmotic solution as a pretreatment of the drying process for beet snacks.

### 3.2. Acidity

Titratable acidity refers to the measure of the total amount of acid present in a solution. Literature values for fresh beetroot titratable acidity range from 0.08% to 0.29% [5,46], while literature values for sugar beet molasses titratable acidity range from 0.84% to 2.57% [47]. In this study, titratable acidity for the fresh beetroot was 0.21%, and for sugar beet molasses, it was 0.82%. Titratable acidity values of the 15 osmotically dehydrated beetroot samples were in the range 1.06–1.52% d.m. (Table 1), which corresponds to 0.24–0.63%, determined according to fresh matter. The increase in the value of titration acidity of OD beetroot compared to the fresh beetroot sample was the result of contact with the medium for dehydration—sugar beet molasses. Sugar beet molasses is a byproduct of sugar production and can vary in acidity. Sugar beet molasses contains various acids, and the predominant ones are organic acids: lactic acid, acetic acid, malic acid, pyrocarbonic acid, oxalic acid, glycolic acid, and citric acid [48]. The acidity of runs 7 and 8 stood out as having significantly (*p* < 0.05) lower values, while run 1 showed the highest acidity value, and there were no significant differences (*p* > 0.05) in acidity between the other samples, regardless of the applied process parameters.

### 3.3. Mineral Content

Concerning the Mg content for runs 1–15, it was in a range from 147.66 to 504.62 mg/100 g (an increase of 3.42 times); the K content was from 7861.18 to 17,968.71 mg/100 g (an increase of 2.28 times); the Na content ranged from 3037.02 to 7038.92 mg/100 g (an increase of 2.32 times), and the Ca from 380.86 to 1706.96 mg/100 g (an increase of 4.48 times). Data in the literature indicate that the average mineral composition of beetroots corresponds to 2825 mg K/L, 439 mg Na/L, 69,3 mg Ca/L, and 259 mg Mg/L [49]. Sugar beet molasses is also very rich in minerals, as has been previously reported. Sauvant et al. [50] reported 3920 mg K, 680–1300 mg Na, 100 mg Ca, and 50–320 mg Mg per 100 g sugar beet molasses. Šuput et al. [51] observed higher values for Ca (666.31 mg/100 g), while other minerals were in similar ranges: 2399.76 mg K, 1046.21 mg Na, and 86.39 mg Mg per 100 g sugar beet molasses. The rich mineral composition of molasses positively affects the mineral composition of the osmotically dehydrated product [51,52].

Based on the presented results, it can be concluded that by varying the parameters of the dehydration procedure, the mineral composition of the product can be modified. The highest mineral intakes were obtained in runs 12, 11, 4, and 8. These runs were correlated to the longest process duration (time = 5 h). On the other hand, the lowest mineral intakes were recorded for runs 5, 6, 9, and 10, because these runs were correlated to the shortest process duration (time = 1 h). These results clearly indicate that the parameter “time” is of crucial importance for the final mineral composition of beetroots, in comparison to the influences of temperature and concentration.

### 3.4. Total Phenols and Flavonoids

The total phenolic contents of beetroot juice, beetroot powder, beetroot chips, and cooked beetroot have been found to be 3.67, 0.51, 0.75, and 2.79 GAE mg/g [53,54], respectively. This suggests that the processing method has an impact on the quantity and quality of the phenolic content [1]. When it came to the treatments, vacuum drying retained more phenolic compounds than hot-air-drying [55]. Xu et al. [56] found that in terms of phenolic content, samples that were freeze- and microwave-dried performed better than samples that were hot-air-dried. In this instance, it was believed that high temperatures were the mechanism causing the phenolic to be destroyed during hot-air-drying. The highest values for flavonoids and phenols in this study were achieved for runs 1, 2, 8, 9, 10, and 13, which were dehydrated mostly at mild temperatures (40 °C) and at medium times (3 h). The obtained results align with the trend in which shorter drying times contribute to the preservation of higher concentrations of phenolic compounds [20].

Novel osmotic solutions can also be utilized to raise the phenolic content of dried fruits and vegetables, as was previously mentioned relative to antioxidant capacity [57,58]. As a rich source of phenolic compounds, such as apigenin, luteolin, kaempferol, chrysin, catechin, chlorogenic acid, gallic acid, p-coumaric acid, vanillic acid, ferulic acid, and syringic acid, sugar beet molasses is one of these solutions, as used in this investigation [59].

### 3.5. Betaine Content

Betaine content values were statistically significantly different, ranging from 1111.45 mg/100 g to 3828.18 mg/100 g. Betaine content in beetroots varies from 750 to 3337 μg/g d.m. and beetroot itself is considered to be a rich source of betaine, containing more than 150 μg/g d.m. [60]. The obtained results exceeded the common values due to the contribution of molasses, since sugar beet molasses contains 5–6 g of betaine per 100 g, representing the most abundant source of betaine [60]. The highest values were recorded in samples 8 and 12 (3828.18 mg/100 g and 3140.25 mg/100 g, respectively), which correspond to the process parameters 60 °C, 70%, 5 h, and 40 °C, 80%, and 5 h, respectively. What they have in common is a maximal duration of the osmotic dehydration process, followed by the high temperatures and concentrations applied. The lowest values were recorded in samples 6 and 9 (1111.45 mg/100 g and 1264.90 mg/100 g, respectively), which correspond to the following process parameters: 20 °C, 70%, 1 h, and 40 °C, 60%, and 1 h. Both runs were applied under the minimum duration of the osmotic dehydration procedure, disregarding, for the analysis, the moderate applied temperatures and concentrations. It can be concluded that the longer the contact of sugar beet molasses with beetroots, the greater will be the increase in betaine content in the osmotically dehydrated product (beetroots).

### 3.6. Antioxidative Activity

Compared to many other fruits and vegetables thought to be rich in antioxidants, beetroot exhibits stronger antioxidant activity. Along with phenolic acids, B vitamins, and flavonoids, betalain has been determined to constitute approximately 50% of the overall antioxidant activity of beetroot [1]. Due to the great sensitivity of betalains to physical parameters (temperature, pH, light, aeration, and water activity) [61], the antioxidant potentials of different preservation strategies may vary depending on these factors [1]. Some authors claim that antioxidant assays show the detrimental effects of drying on food matrices because drying time appears to be a critical process variable influencing the antioxidant content of dried fruits and vegetables, while the impact of drying temperature cannot be neglected [20].

For instance, the antioxidant capability is impacted by the widely employed convective airflow drying process. It entails the prolonged use of high temperatures, which inhibits antioxidant activity and results in unfavorable alterations [62,63]. Antioxidant assays can therefore be useful for some parameter assessments that are part of a drying optimization. Osmotic dehydration is regarded as a mild preservation method which doesn’t involve excessive heat. The temperature range of 20 °C to 60 °C in this experiment had an impact on the *DPPH* values, which were in the range of 2.84 IC_50_ (mg/mL)–8.34 IC_50_ (mg/mL), and the *ABTS* values, which were in the range of 0.80 IC_50_ (mg/mL)–3.14 IC_50_ (mg/mL), suggesting substantial antioxidative activity. This activity results from sugar beet molasses serving as an OD medium since sugar beet molasses is a rich source of antioxidants, one which enhances a food’s safety and health benefits when added [64,65]. The obtained results are consistent with previous research using sugar beet molasses [44,66], which determined that during OD certain phenolic compounds from the molasses diffuse into the plant tissue, contributing to the overall antioxidative potential.

Knežević et al. [66] and Guimarães et al. [67] highlighted that higher temperature and longer processing time led to an increase in antioxidant capacity in samples osmotically treated using sugar beet molasses. On the other hand, Lopez et al. [68] stated that 50 °C drying temperature and its longer associated time are more harmful to phenolics than 60 and 70 °C. Regarding the effects of temperature and time in this work, the lowest values in the *DPPH* and *ABTS* tests, observations which indicate high antioxidative potential, were obtained in runs 4, 8, and 12, in which the maximum process parameters were applied, i.e., the highest values for temperature, concentration, and time.

### 3.7. Process Optimization

The standard score (SS) was obtained by summing the normalized scores for each variable (*DMC*, *WL*, *SG*, Mg, K, Na, Ca, *DPPH*, *ABTS*, flavonoid and phenol content, acidity, and betaine content). By maximizing the SS function, the optimal processing parameters (*T*, *Conc*, and *t*), as well as the optimal values for *DMC*, *WL*, *SG*, Mg, K, Na, Ca, *DPPH*, *ABTS*, flavonoid and phenol content, acidity, and betaine content are determined. When the SS function approaches a value of 1, it indicates a stronger likelihood that the tested processing parameters are optimal.

**Figure 1:**
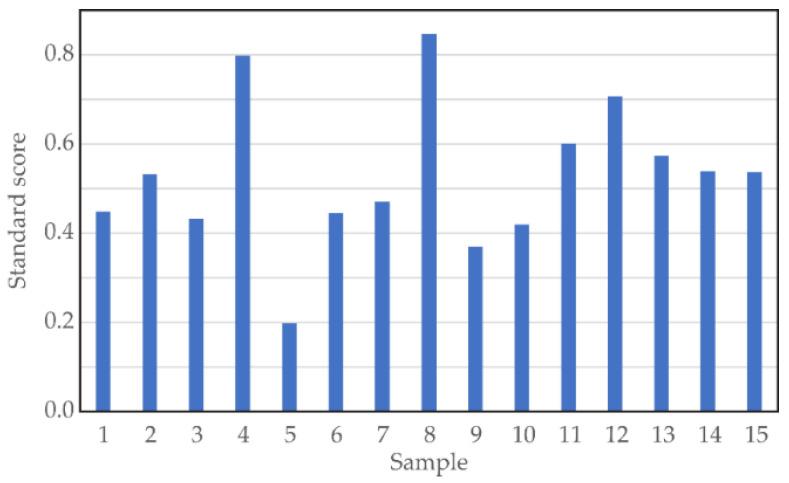
Standard scores for 15 samples during osmotic dehydration.

According to the results of the standard score analysis (Figure 1), the highest SS value was obtained for sample 8 (*T* = 60 °C, *Conc* = 70%, *t* = 5 h), with output variable values equal to *DMC* = 50.149%; *WL* = 0.708 g/g_f.s._; *SG* = 0.050 g/g_f.s._; Mg = 158.18 mg/kg; K = 15,268.18 mg/kg; Na = 4729.92 mg/kg; Ca = 1706.96 mg/kg; *DPPH* = 2.84 IC_50_ (mg/mL); *ABTS* = 0.80 IC_50_ (mg/mL); flavonoid content = 145.39 mg ECA/100 g d.m.; phenol content = 646.45 mg eq.GA/100 g d.m.; acidity = 1.06% d.m.; and betaine content = 3828.18 mg/100 g d.m. The obtained SS value reached 0.847.

### 3.8. Correlation Analysis

**Figure 2:**
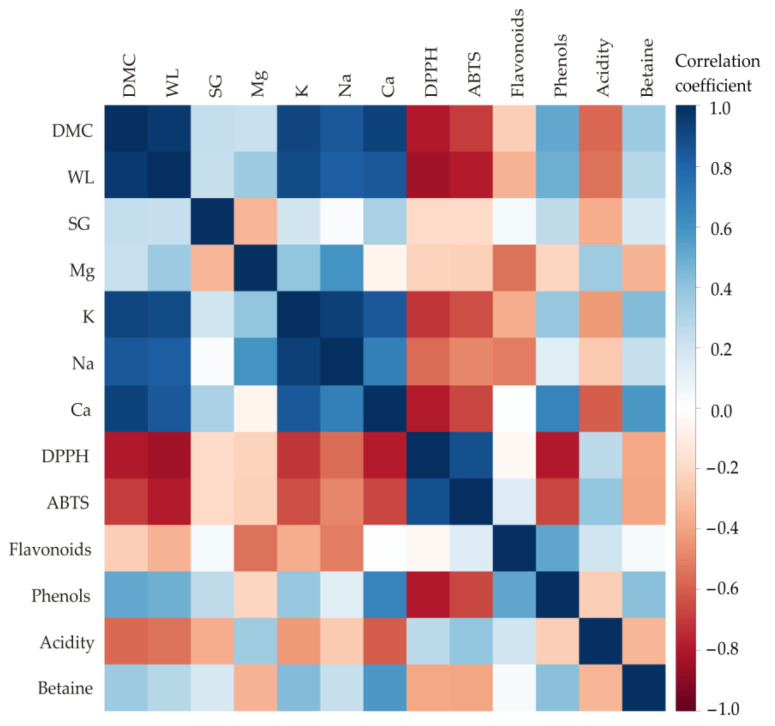
Color graph depicting correlation among observed responses. The color of the square corresponds to the correlation coefficients: blue indicates a positive correlation, while red denotes a negative correlation [69].

In Figure 2, a color correlation graph is presented illustrating the relationships among all examined outputs for characterization of the beetroot samples. Significant correlations (*p* < 0.05) were identified across most of the analyzed responses.

Based on the correlation analysis (Table 2), significant positive associations were found between *DMC* and *WL*, indicating that as DMC increases, WL also tends to increase significantly (*r* = 0.958, *p* < 0.001). Similar strong positive correlations were observed between DMC and K content (*r* = 0.912, *p* < 0.001) and Ca content (*r* = 0.930, *p* < 0.001). Additionally, strong negative correlations were noted between *WL* and K content, reaching *r* = 0.901, with statistical significance at the *p* < 0.001 level, indicating that as water loss increases, potassium content tends to decrease significantly. Furthermore, positive correlations emerged between K and Na content levels (*r* = 0.936, *p* < 0.001), suggesting that as potassium content increases, sodium content also tends to increase significantly. Significant correlations were also observed between Na content and *DMC* (*r* = 0.841; *p* < 0.001) and Na content and *WL* (*r* = 0.830; *r* = −0.001), indicating that as sodium content increases, dry matter content also tends to increase significantly. Positive correlations were identified between K and Ca content (*r* = 0.845, *p* < 0.001). A positive correlation was observed between *DPPH* and *ABTS* (*r* = 0.873, *p* < 0.001). Significantly negative correlations were noticed between *DPPH* and *WL* (*r* = −0.829, *p* < 0.001), indicating that as antioxidant capacity measured by DPPH increases, water content tends to decrease significantly, and also noticed between *DPPH* and phenol content (*r* = −0.800, *p* < 0.001), indicating that as antioxidant capacity measured by DPPH increases, phenol content tends to decrease significantly.

### 3.9. Cluster Analysis

**Figure 3:**
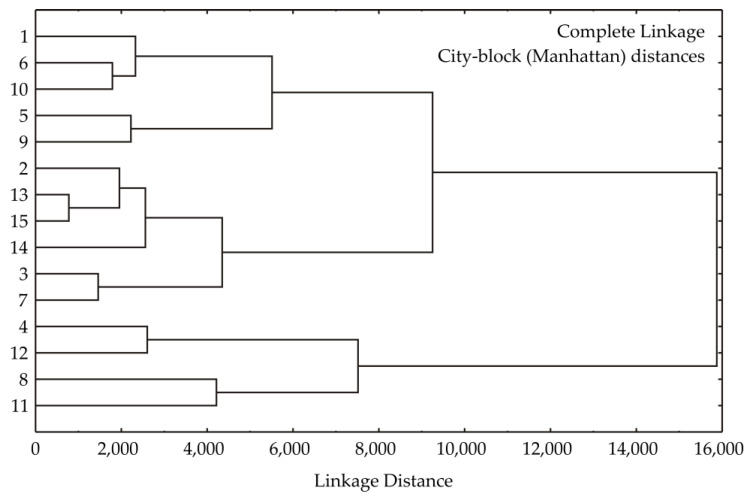
Cluster analysis of observed sections.

Figure 3 illustrates the results of a cluster analysis conducted on the observed samples using the complete linkage algorithm and city block (Manhattan) distances to measure sample proximity. City block distances, representing the average difference between sample dimensions, are plotted on the abscissa axis. The linkage distance between main clusters was substantial, at around 16,000. The dendrogram generated from the analysis revealed four main clusters. The analysis, based on data regarding *DMC*, *WL*, *SG*, Mg, K, Na, Ca, *DPPH*, *ABTS*, flavonoid and phenol content, acidity, and betaine content, aimed to highlight similarities among observed parameters. Cluster 1 comprised sites 1 (*T* = 20 °C, *Conc* = 60%, *t* = 3 h), 6 (*T* = 60 °C, *Conc* = 70%, *t* = 1 h), and 10 (*T* = 40 °C, *Conc* = 80%, *t* = 1 h), while cluster 2 included sites 5 (*T* = 20 °C, *Conc* = 70%, *t* = 1 h) and 9 (*T* = 40 °C, *Conc* = 60%, *t* = 1 h). Cluster 3 consisted of sites 2 (*T* = 60 °C, *Conc* = 60%, *t* = 3 h), 13, 14, 15 (*T* = 40 °C, *Conc* = 70%, *t* = 3 h), 3 (*T* = 20 °C, *Conc* = 80%, *t* = 3 h), and 7 (*T* = 20 °C, *Conc* = 70%, *t* = 5 h), while cluster 4 consisted of samples 4 (*T* = 60 °C, *Conc* = 80%, *t* = 3 h), 8 (*T* = 60 °C, *Conc* = 70%, *t* = 5 h), 11 (*T* = 40 °C, *Conc* = 60%, *t* = 5 h), and 12 (*T* = 40 °C, *Conc* = 80%, *t* = 5 h).

Samples in clusters are grouped according to similar examined properties (output parameters). What is similar for the samples of cluster 1 are low values of DMC, WL, and SG and low contents of minerals and betaine, as well as moderate content levels of phenols and flavonoids. The highest values for osmotic dehydration parameters (DMC, WL, and SG), mineral content, and antioxidative content and potential were observed for cluster 4 samples. Samples grouped in clusters 2 and 3 have very similar properties—mostly moderate with a tendency that the characteristics of cluster 2 samples lean towards the characteristics of the cluster 1 samples, while the samples of cluster 3 have more characteristics similar to the samples of cluster 4.

### 3.10. Principal Component Analysis (PCA)

**Figure 4:**
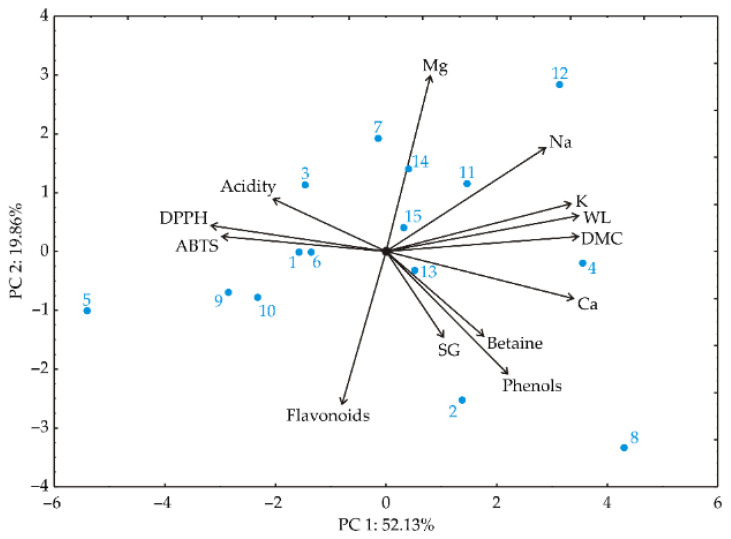
PCA ordination of phenolic content, projection in PC1–PC2 plane. Labels 1–15 refer to tested sample runs.

Principal Component Analysis (PCA) was used to explore the relationships between different samples. The results of the PCA analysis are depicted in Figure 4. The proximity of the spots in the PCA graphic indicates similarity in patterns. The direction of the vectors in the factor space reveals the trends of the observed variables, while the length of the vectors represents the strength of the correlation between the fitting value and the variable [70]. By examining Figure 4, one can efficiently determine the correlations between the contents of various variables, as the angles between corresponding variables reflect the degree of correlation, with smaller angles corresponding to stronger correlations.

The first two PCs demonstrated 72.0% of the total variance in the recorded data. The first PC explained 52.13%, and the second 19.87%, of the total variance between the collected data.

The PCA analysis of beet samples (depicted in Figure 4) revealed that the first two principal components accounted for 72.0% of the total variability among the 13 parameters (*DMC*, *WL*, *SG*, Mg, K, Na, Ca, *DPPH*, *ABTS*, flavonoid and phenol content, acidity, and betaine content). In this analysis, *DMC* (contributing 13.68% to the total variance); *WL* (13.61%); and the contents of K (12.87%), Na (9.44%), and Ca (12.89%) showed a positive influence on the first principal component (PC1). Conversely, *DPPH* (11.32%) and *ABTS* (9.96%) values had negative impacts on the calculation of PC1. Meanwhile, the content levels of Mg (29.60% of total variance) and Na (10.30%) exhibited positive effects on the second principal component (PC2). In contrast, *SG* (7.12%), and the content levels of flavonoids (22.49%) and phenols (14.25%) displayed negative influences on the calculation of PC2.

### 3.11. Artificial Neural Network Model

The artificial neural network model’s structure and results heavily rely on the initial assumptions for matrix parameters (biases and weights). These assumptions are critical for fitting the model to the actual experimental data. The performance of the model is also influenced by the number of neurons in the hidden layer. To address this, 100,000 runs with randomized topologies eliminated random correlations from initial assumptions and weight initialization. The model achieved the highest *r*^2^ value with nine hidden neurons. Each ANN model underwent training for 100 epochs. The training accuracy increased with each training cycle until it reached a nearly constant value around the 50th to 60th epoch. Training for more than 60 epochs could potentially lead to significant overfitting, while 60 epochs proved sufficient for achieving high model accuracy without the risk of overfitting.

The optimized neural network models demonstrated strong generalization for the experimental data, accurately predicting output based on input parameters (Table 2). The ANN models used 3–8 neurons to achieve high *r*^2^ values (the *r*^2^ value ranged from 0.529 to 0.998 in the training cycle). The artificial neural network models exhibited good accuracy in predicting the experimental variables across a diverse range of process variables.

The model feature fit was examined and is presented in Table 2. The results show that the ANN models had minor lack of fit for some tests, which implies that the models satisfactorily predicted the values of the analyzed parameters. The obtained *r*^2^ for *DMC, WL, SG*, Mg, K, Na, Ca, *DPPH*, *ABTS*, flavonoid and phenol content, acidity, and betaine content prediction suggest that the variation was accurately evaluated and that the data fit adequately to the suggested model.

The developed ANN models were evaluated using relative percent error (ranging from 3.133 to 56.235), root mean square error (*RMSE*) values between 0.009 to 2179.919, and average absolute relative deviation (*AARD*) values spanning from 0.094 to 24,446.515. These assessments, outlined in Table 3, demonstrate the statistical significance of the ANN model and its alignment with experimental outcomes. Furthermore, the residual data analysis was performed on the model developed, as an additional model test. The skewness and kurtosis values in Table 3 offer crucial insights into the data distribution, influencing preprocessing steps and model selection for predictive analysis. The negative skewness values and relatively lower kurtosis indicate a left-skewed distribution, suggesting less pronounced values. These metrics play a key role in describing data shape, providing valuable insights into symmetry, tails, and the presence of outliers or extreme values [71].

### 3.12. Global Sensitivity Analysis—Yoon’s Interpretation Method

**Figure 5:**
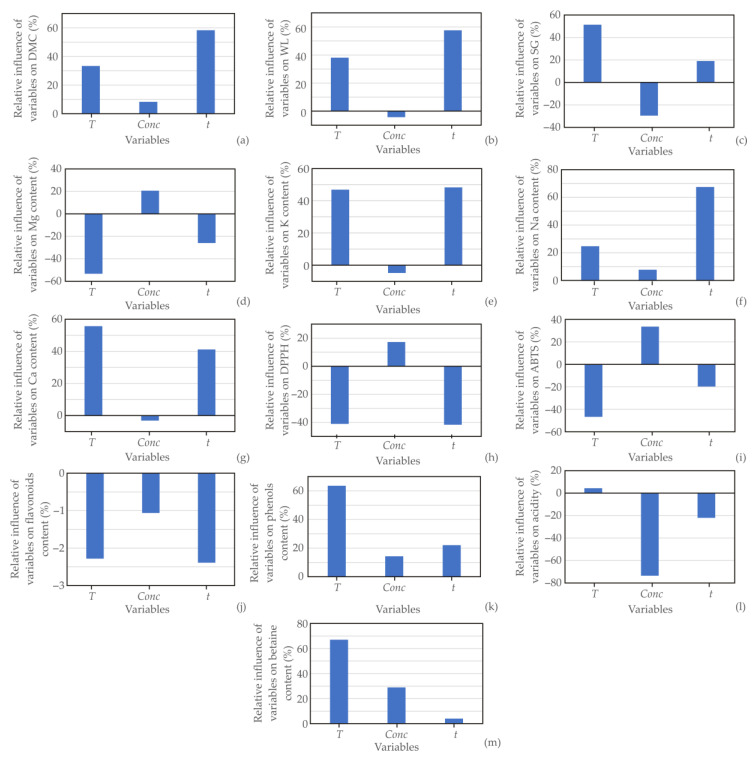
The relative importance of the temperature, osmotic dehydration time, and molasses concentration for (**a**) *DMC*, (**b**) *WL*, (**c**) *SG*, (**d**) Mg content, (**e**) K content, (**f**) Na content, (**g**) Ca content, (**h**) *DPPH*, (**i**) *ABTS*, (**j**) flavonoids, (**k**) phenols, (**l**) acidity, and (**m**) betaine content.

Improved further design as a consequence of understanding a system’s behavior is the main advantage of global sensitivity analysis (GSA). Yoon’s interpretation of (GSA) identifies the main influential parameters and their interaction effects. In this section, Yoon’s interpretation method was employed to investigate the influence of input variables on the relative importance of *T*, *Conc*, and *t* (Figure 5). *T* and *t* emerged as the most influential positive parameters, impacting *DMC*, *WL*, *SG*, and the content of K, Na, and Ca, as well as the phenol and betaine content levels. The concentration of molasses positively affected *DMC,* Na and betaine content levels, and negatively affected *WL* and *SG*, as well as K, Ca, and phenol content. This conclusion agrees with the PCA analysis.

Contrarily, *T* and *t* variables contributed negatively to the content levels of Mg. *T* and *t* negatively influenced *DPPH* and *ABTS*, as well as flavonoid content levels. The acidity was most negatively affected by *Conc*, while the influences of *T* and *t* were negligible. The processing time was identified as the most influential factor among the observed variables.

Recent studies have employed Yoon’s interpretation method to investigate different food materials and drying techniques. Lončar et al. found that the addition of osmotically pre-treated apple powder and the osmotic treatment of sugar beet molasses were crucial factors influencing muffin samples’ chemical compositions and texture [72]. Conversely, Šovljanski et al. utilized global sensitivity analysis to explore the effects of various drying methods (including convective drying, lyophilization, and osmotic dehydration in sugar beet molasses) and sweet potato variety selection on multiple output variables, such as total phenolic contents, antioxidant capabilities, pharmacological activity, color properties, chemical composition, and mineral content [73]. Additionally, Demir et al. applied Yoon’s global sensitivity equation to assess the impacts of drying conditions (temperature and time) on specific energy consumption, highlighting processing time as the more influential parameter compared to drying temperature [74].

### 3.13. Storage Stability

The beetroots were osmotically dehydrated under selected optimal osmotic drying conditions to determine quality and stability over 4 weeks. Considering the complex system of highly valuable nutrients in both beetroot and sugar beet molasses, we applied a biopolymer coating synthesized from camelina cake (CSoC-based coating) to half of the samples to determine the effectiveness of the biofilm in preserving antioxidant activity and microbiological safety. Our previous research has confirmed that CSoC films have pronounced antioxidant and antimicrobial potential [42]. Therefore, we endeavored to ascertain whether these attributes persist when the CSoC coating is administered to a particular product. So far, there have been many examples of using the application of edible coatings as a way of preserving, or improving, the antioxidant compounds present in fruits or vegetables [75,76,77]. We chose osmotically dehydrated beets precisely because of their high-quality but sensitive composition. Table 4 shows the quality results of osmotically dehydrated beetroots, both with and without coating—over the storage period.

Acidity values were in the range of 0.85% d.m.–1.30% d.m. Over time, a decrease in acidity was observed in both uncoated and coated samples of osmotically dehydrated beetroots. These findings are in agreement with the results of quality losses in fresh-cut red beets [5].

The results revealed that the total phenols and total flavonoids of osmotically dehydrated beetroots (coated or not) decreased gradually with the increase of the storage period. This decrease was statistically significant. Total phenol content decreased from 1016.05 to 458.09 mg eq.GA/100 g d.m. for osmotically dehydrated uncoated samples, and from 1063.14. to 571.29 mg eq.GA/100 g d.m. for osmotically dehydrated coated samples. Total flavonoid content decreased from 238.80 to 63.37 mg ECA/100 g d.m. for osmotically dehydrated uncoated samples, and from 257.43. to 105.38 mg ECA/100 g d.m. for osmotically dehydrated coated samples. Consistently, at each designated sampling time, the beet samples coated with CSoC-based coating consistently exhibited higher total phenol and flavonoid content than their counterparts without coating. According to the literature, six flavonoids (quercitrin, isoquercetin, catechic acid, epicatechin, rutin, and isorhamnetin), three hydroxybenzoic acids (salicylic acid, proto-catechuic acid, and p-hydroxybenzoic acid) and one hydroxycinnamic acid (sinapic acid) have been identified in CSoC [78]. The synergism between polyphenols and water-soluble amino acids and their derivatives indicates the high level of antioxidant activity of biopolymer coating based on CSoC [42]. So, the naturally present active components in the camelina-based coating were associated with higher phenolic and flavonoid values in coated beetroot samples during the entire storage period, compared to uncoated beetroot samples.

Although high betaine levels were achieved by the dehydration with sugar beet molasses, which is considered a raw material for the extraction of betaine [79,80], the results pointed out a gradual decline in betaine content during beet storage, which is due to its instability. The obtained results are in agreement with the investigations of Viskelis et al. [10] and Akan et al. [5]. The betaine concentration achieved in the original dehydrated beet sample decreased from 3175.46 to 2785.48 mg/100 g d.m., while the dehydrated beet sample coated with CSoC base biopolymer evinced a decrease from 3343.13 to 3082.87 mg/100 g d.m.

*DPPH* and *ABTS* tests indicated a slight reduction of antioxidant properties over the storage time. Akan et al. [5] suggest that the reason for this may be a decrease in betaine concentration over time. In addition to the decrease in betaine content, the reduction in antioxidant activity can be attributed to a concurrent decrease in the concentrations of phenols and flavonoids. On day 0 of the storage stability test, the *DPPH* value at the beginning was 1.56 IC_50_ (mg/mL) for uncoated, and 1.37 IC_50_ (mg/mL) for coated, beetroot samples. Both samples had the lowest values on the 14th day of storage, but eventually decreased to 2.22 IC_50_ (mg/mL) for the uncoated sample and 1.295 IC_50_ (mg/mL) for the coated sample. During the whole storage period, the osmotically dehydrated beetroot sample coated with a CSoC-based biopolymer had lower values compared to the uncoated sample. *ABTS* values were also higher for the uncoated sample compared to the coated one during the whole storage period, except on the 21st day, when the uncoated sample had an IC_50_ of 0.45, whereas the coated sample had an IC_50_ of 0.53. The decreasing trend is not so pronounced as in the *DPPH* test, since starting and final *ABTS* values were very similar in the case of both samples. It can be concluded that, similar to the high content of phenols and flavonoids values, the lower *DPPH* and *ABTS* test values indicate a higher antioxidant potential in samples of osmotically dehydrated beets coated with a CSoC biopolymer film. Therefore, the presence of active compounds from the biopolymer coating and the synergism with the active compounds present in the osmotically dehydrated beet affected the lower values of *DPPH* and *ABTS*, compared to the uncoated samples.

Preserving food to extend its shelf life is a central preoccupation of the food industry. One of the approaches to reducing the microbial load is the osmotic dehydration process, which can be used to develop new fruit and vegetable products due to its ability to improve the sensory and nutritional properties of fresh products, as well as to extend the shelf life of the final product [23,81,82]. With that in mind, this part of the study was initiated to evaluate the microbiological quality of osmotically dehydrated beetroots, as well as osmotically dehydrated beetroots coated with CSoC biopolymer, in order to assess its antimicrobial effects.

As presented in Table 5, the microbiological analysis showed that *Escherichia coli*, *Enterobacteriaceae*, *Salmonella* spp., *Listeria monocytogenes*, *Clostridium perfringens*, and the total number of yeast and molds were not detected in any of the tested samples. These findings are in agreement with other studies, which showed that osmotic dehydration treatments have a highly reductive effect on most microorganisms [83,84]. However, based on the data obtained by the ISO method, it was found that the total number of microorganisms of osmotically dehydrated beets and osmotically dehydrated beets coated with *Camelina sativa* biopolymer at day 0 amounted to 2600 CFU/g and 2400 CFU/g, respectively. Nevertheless, it should be emphasized that the total number of microorganisms in both samples decreased during the storage period, as shown in Table 5. The specific investigation conducted by Akharume et al. [81] also revealed that storage time did have significant effects on microbial growth and reduction. Additionally, regarding the samples coated with CSoC biopolymer in the current study, we observed its inhibitory effect on microbial growth (Table 1), which is consistent with our previous research, in which *Camelina sativa* possessed antimicrobial properties [42]. The antimicrobial activity of *Camelina sativa* may be due to the presence of certain compounds, such as phenols, polyphenols, and glucosinolates, in camelina seeds, as reported before by [85]. Overall, based on the results of the microbiological profile of osmotically dehydrated beets, it was shown that osmotic dehydration is one of the methods suitable for increasing the shelf life of vegetables, as well as the application of CSoC biopolymer coating being a means of additional protection for osmotically treated beetroots.

It is certain that storage at a low temperature also contributed to good quality preservation, which is in accordance with studies of Pandey et al. [86] and Caldas-Cueva et al. [87].

## 4. Conclusions

This study highlights the influence of process parameters (time, temperature, and concentration) on the efficiency of the osmotic dehydration of beetroots and the improvement of the nutritional and antioxidant properties of the treated samples. The results suggest that careful manipulation of process conditions can significantly influence the quality of osmotically dehydrated products. As a result of applying high temperatures and concentrations, as well as the longest osmotic dehydration time, during the osmotic dehydration process, sample 8 (temperature of 60 °C, molasses concentration of 70%, and processing time of 5 h) stood out for the majority of the beetroot quality parameters tested. The obtained quality values for sample 8 were: *DMC* = 50.149%; *WL* = 0.708 g/g_f.s._; *SG* = 0.050 g/g_f.s._; Mg = 158.18 mg/kg; K = 15,268.18 mg/kg; Na = 4729.92 mg/kg; Ca = 1706.96 mg/kg; *DPPH* = 2.84 IC_50_ (mg/mL); *ABTS* = 0.80 IC_50_ (mg/mL); flavonoid content = 145.39 mg ECA/100 g d.m.; phenol content = 646.45 mg eq.GA/100 g d.m.; acidity = 1.06% d.m.; and betaine content = 3828.18 mg/100 g d.m. Furthermore, the stability of the dehydrated beetroots was investigated over the 28 days following the application of ideal dehydration conditions. Half of the dried samples were coated with an edible biopolymer coating based on *Camelina sativa* oilcake, while the other half of the samples remained uncoated. All assessed quality parameters were evaluated as better when the osmotically dehydrated beets were additionally coated, compared to the parameters of uncoated samples. To maintain the quality of osmotically dehydrated beetroots during storage, this research emphasizes the significance of the application of a biopolymer coating.

## Figures and Tables

**Table 1:** Box–Behnken experimental design with process variables and obtained responses.

No.	*T*	*Conc*	*t*	*DMC* (%)	*WL* (g/g_f.s._)	*SG* (g/g_f.s._)	Mg (mg/kg)	K (mg/kg)	Na (mg/kg)	Ca (mg/kg)
1	20	60	3	26.737 ± 0.134 ^c^	0.536 ± 0.006 ^e^	0.005 ± 0.000 ^ab^	375.44 ± 0.37 ^cd^	10,564.15 ± 22.48 ^abc^	3798.08 ± 19.10 ^bc^	595.18 ± 3.52 ^bc^
2	60	60	3	35.614 ± 0.270 ^e^	0.606 ± 0.006 ^g^	0.032 ± 0.002 ^h^	202.82 ± 0.19 ^a^	9050.28 ± 7474.60 ^a^	4007.28 ± 22.75 ^bcd^	1080.12 ± 9.00 ^f^
3	20	80	3	29.572 ± 0.178 ^d^	0.571 ± 0.005 ^f^	0.009 ± 0.000 ^bc^	354.46 ± 0.74 ^bcd^	11,477.97 ± 41.83 ^abc^	4178.06 ± 15.76 ^cde^	689.61 ± 8.58 ^d^
4	60	80	3	43.583 ± 0.085 ^g^	0.679 ± 0.006 ^i^	0.032 ± 0.000 ^f^	344.59 ± 0.27 ^bcd^	17,968.71 ± 113.41 ^d^	5893.59 ± 29.05 ^g^	1324.41 ± 10.99 ^h^
5	20	70	1	16.574 ± 0.129 ^a^	0.333 ± 0.000 ^a^	0.011 ± 0.000 ^bcd^	147.66 ± 0.17 ^a^	7861.18 ± 52.01 ^a^	3048.38 ± 25.06 ^a^	380.86 ± 1.29 ^a^
6	60	70	1	26.790 ± 0.183 ^c^	0.508 ± 0.003 ^d^	0.016 ± 0.000 ^d^	328.24 ± 0.69 ^bcd^	10,540.82 ± 48.81 ^abc^	3737.85 ± 23.29 ^bc^	609.54 ± 7.36 ^bcd^
7	20	70	5	36.969 ± 0.260 ^ef^	0.355 ± 0.008 ^a^	0.025 ± 0.000 ^e^	349.60 ± 0.79 ^bcd^	11,961.57 ± 3.68 ^abc^	4674.94 ± 40.07 ^ef^	683.11 ± 4.16 ^cd^
8	60	70	5	50.149 ± 0.626 ^h^	0.708 ± 0.004 ^j^	0.050 ± 0.000 ^g^	158.18 ± 0.11 ^a^	15,268.18 ± 82.90 ^bcd^	4729.92 ± 29.07 ^ef^	1706.96 ± 7.75 ^i^
9	40	60	1	22.921 ± 0.053 ^b^	0.449 ± 0.001 ^b^	0.009 ± 0.000 ^bc^	319.23 ± 1.25 ^bc^	8383.86 ± 65.78 ^a^	3037.02 ± 20.93 ^a^	424.39 ± 5.17 ^a^
10	40	80	1	23.273 ± 0.211 ^b^	0.479 ± 0.001 ^c^	0.002 ± 0.000 ^a^	307.67 ± 0.35 ^b^	9657.91 ± 68.56 ^ab^	3462.99 ± 11.45 ^ab^	572.99 ± 3.53 ^b^
11	40	60	5	38.287 ± 0.226 ^f^	0.633 ± 0.005 ^h^	0.036 ± 0.000 ^f^	377.15 ± 1.19 ^d^	16,112.23 ± 115.48 ^cd^	5339.88 ± 31.25 ^fg^	991.61 ± 10.36 ^f^
12	40	80	5	47.999 ± 0.500 ^h^	0.710 ± 0.002 ^j^	0.038 ± 0.000 ^f^	504.62 ± 0.72 ^e^	18,174.63 ± 160.90 ^d^	7038.92 ± 38.62 ^h^	1182.05 ± 9.28 ^g^
13	40	70	3	31.357 ± 1.462 ^d^	0.584 ± 0.017 ^fg^	0.015 ± 0.005 ^cd^	350.30 ± 42.53 ^bcd^	13,083.26 ± 560.93 ^abcd^	4497.41 ± 487.14 ^de^	794.38 ± 66.07 ^e^
14	40	70	3	31.087 ± 1.526 ^d^	0.583 ± 0.018 ^fg^	0.015 ± 0.005 ^cd^	349.73 ± 41.21 ^bcd^	13,106.91 ± 556.89 ^abcd^	4504.91 ± 476.96 ^de^	794.58 ± 65.25 ^e^
15	40	70	3	31.188 ± 1.694 ^d^	0.586 ± 0.015 ^fg^	0.015 ± 0.005 ^cd^	349.05 ± 41.91 ^bcd^	13,003.68 ± 487.00 ^abcd^	4497.81 ± 514.57 ^de^	800.30 ± 63.66 ^e^
**No.**	***T***	***Conc***	***t***	***DPPH*** **(IC_50_ (mg/mL))**	***ABTS*** **(IC_50_ (mg/mL))**	**Flavonoids** **(mg ECA/100 g d.m.)**	**Phenols** **(mg eq.GA/100 g d.m.)**	**Acidity** **(% d.m.)**	**Betaine Content** **(mg/100 g d.m)**	
1	20	60	3	4.51 ± 0.01 ^bc^	1.92 ± 0.12 ^bcd^	135.50 ± 5.47 ^efg^	450.38 ± 68.26 ^cd^	1.52 ± 0.23 ^b^	1446.72 ± 15.32 ^ab^	
2	60	60	3	4.79 ± 0.01 ^c^	1.26 ± 0.04 ^abcd^	113.83 ± 13.35 ^bcd^	459.41 ± 65.74 ^cd^	1.12 ± 0.002 ^ab^	2475.85 ± 9.38 ^e^	
3	20	80	3	6.63 ± 0.01 ^f^	1.98 ± 0.01 ^bcd^	106.98 ± 0.63 ^abcd^	343.57 ± 9.46 ^abc^	1.39 ± 0.01 ^bcd^	1713.08 ± 40.62 ^bc^	
4	60	80	3	4.23 ± 0.02 ^bc^	0.91 ± 0.06 ^a^	119.17 ± 2.18 ^cde^	488.18 ± 10.22 ^d^	1.15 ± 0.14 ^ab^	2975.39 ± 514.49 ^f^	
5	20	70	1	8.34 ± 0.02 ^g^	3.14 ± 0.24 ^e^	123.32 ± 7.38 ^def^	301.56 ± 30.59 ^a^	1.42 ± 0.18 ^ab^	1111.45 ± 61.40 ^a^	
6	60	70	1	5.44 ± 0.02 ^d^	1.44 ± 0.08 ^abcd^	112.84 ± 2.61 ^bcd^	405.61 ± 6.20 ^abcd^	1.34 ± 0.001 ^ab^	1861.46 ± 28.84 ^bc^	
7	20	70	5	5.79 ± 0.01 ^de^	1.61 ± 0.23 ^abcd^	85.88 ± 3.29 ^a^	311.83 ± 4.74 ^ab^	1.07 ± 0.01 ^a^	1976.45 ± 15.57 ^cd^	
8	60	70	5	2.84 ± 0.01 ^a^	0.80 ± 0.08 ^a^	145.39 ± 2.15 ^g^	646.45 ± 14.07 ^e^	1.06 ± 0.003 ^a^	3828.18 ± 30.16 ^g^	
9	40	60	1	5.72 ± 0.01 ^de^	1.99 ± 0.89 ^cd^	142.77 ± 5.13 ^fg^	468.44 ± 6.94 ^d^	1.39 ± 0.07 ^ab^	1264.90 ± 28.85 ^a^	
10	40	80	1	6.14 ± 0.01 ^ef^	2.09 ± 0.57 ^d^	139.37 ± 2.84 ^efg^	457.81 ± 11.45 ^cd^	1.41 ± 0.14 ^ab^	1484.54 ± 16.16 ^ab^	
11	40	60	5	4.78 ± 0.01 ^c^	1.30 ± 0.02 ^abcd^	102.53 ± 0.83 ^abcd^	455.47 ± 9.26 ^cd^	1.21 ± 0.08 ^ab^	2386.31 ± 3.67 ^de^	
12	40	80	5	4.16 ± 0.01 ^b^	1.43 ± 0.06 ^abcd^	93.36 ± 1.95 ^ab^	400.25 ± 47.17 ^abcd^	1.32 ± 0.00 ^ab^	3140.25 ± 24.36 ^f^	
13	40	70	3	4.75 ± 0.42 ^c^	1.13 ± 0.05 ^abc^	101.40 ± 12.03 ^abc^	429.71 ± 52.07 ^bcd^	1.27 ± 0.09 ^ab^	2312.04 ± 42.95 ^de^	
14	40	70	3	4.74 ± 0.41 ^c^	1.11 ± 0.04 ^ab^	102.07 ± 11.77 ^abc^	482.18 ± 84.29 ^d^	1.34 ± 0.35 ^ab^	2220.13 ± 38.11 ^de^	
15	40	70	3	4.77 ± 0.42 ^c^	1.13 ± 0.05 ^abc^	101.03 ± 11.59 ^abc^	428.64 ± 54.17 ^bcd^	1.26 ± 0.09 ^ab^	2170.54 ± 54.22 ^de^	

^a–g^ Means in the same column with different superscripts are statistically different (*p* ≤ 0.05). In the table, *DMC*—dry matter content (%); *WL*—water loss (g/g_f.s._); and *SG*—solid gain (g/g_f.s._). The relevant units of measurement are as follows: Mg (mg/kg); K (mg/kg); Na (mg/kg); Ca (mg/kg); *DPPH* (IC_50_ (mg/mL)), *ABTS* (IC_50_ (mg/mL)); Flavonoids (mg ECA/100 g d.m.); Phenols (mg eq.GA/100 g d.m.); Acidity (% d.m.); and Betaine content (mg/100 g d.m.).

**Table 2:** Performance of ANN models for observed parameters.

Parameters	Net Name	Performance			Error			Training Algorithm	Activation	
		Train	Test	Valid	Train	Test	Valid		Hidden	Output
*DMC*	MLP 3-4-1	0.930	0.989	1.000	3.477	0.643	7.516	BFGS 11	Log.	Log.
*WL*	MLP 3-8-1	0.838	0.807	1.000	0.001	0.001	0.000	BFGS 4	Log.	Exp.
*SG*	MLP 3-4-1	0.998	0.659	0.998	0.000	0.000	0.000	BFGS 18	Tanh	Identity
Mg	MLP 3-3-1	0.781	0.141	1.000	739.498	804.754	2067.172	BFGS 22	Tanh	Identity
K	MLP 3-8-1	0.746	0.956	1.000	789,630.298	933,408.202	6,993,799.807	BFGS 5	Tanh	Exp.
Na	MLP 3-3-1	0.866	0.931	1.000	33,912.074	31,890.460	1,021,189.947	BFGS 4	Identity	Identity
Ca	MLP 3-6-1	0.760	0.408	1.000	63,490.819	13,155.770	69,075.745	BFGS 2	Tanh	Log.
*DPPH*	MLP 3-4-1	0.885	0.756	1.000	0.515	0.044	0.518	BFGS 2	Tanh	Exp.
*ABTS*	MLP 3-3-1	0.529	0.967	0.997	0.233	0.025	0.130	BFGS 1	Exp.	Tanh
Flavonoids	MLP 3-7-1	0.660	0.759	1.000	58.449	163.423	107.098	BFGS 20	Exp.	Tanh
Phenols	MLP 3-8-1	0.592	0.482	1.000	4631.590	3800.579	1554.333	BFGS 2	Exp.	Log.
Acidity	MLP 3-3-1	0.967	0.983	0.360	0.000	0.000	0.014	BFGS 259	Tanh	Exp.
Betaine	MLP 3-4-1	0.729	0.899	0.972	82,548.984	72,414.239	35,561.058	BFGS 25	Log.	Identity

Error function was the sum of squares (SOS).

**Table 3:** The “goodness of fit” tests for the developed ANN model.

	*χ* ^2^	*RMSE*	*MBE*	*MPE*	*SSE*	*AARD*	r^2^	Skew	Kurt	Mean	StDev	Var
*DMC*	9.30	2.93	1.13	7.37	83.38	50.21	0.93	0.55	−0.72	0.98	2.64	6.95
*WL*	0.00	0.04	0.00	7.11	0.02	0.54	0.86	−0.14	−0.27	0.00	0.04	0.00
*SG*	0.00	0.01	0.00	56.23	0.00	0.09	0.99	0.22	−0.99	0.00	0.01	0.00
Mg	2.5 × 10^3^	48.47	8.59	13.73	25,466.62	696.18	0.76	−0.05	0.60	7.45	46.07	2.1 × 10^3^
K	5.1 × 10^6^	2.2 × 10^3^	536.56	14.02	5.0 × 10^7^	2.4 × 10^4^	0.62	0.78	−0.02	465.02	2.0 × 10^3^	4.2 × 10^6^
Na	5.8 × 10^5^	730.06	264.45	9.16	5.3 × 10^6^	6.5 × 10^3^	0.66	1.52	2.17	229.19	662.30	4.4 × 10^5^
Ca	1.4 × 10^5^	354.77	68.23	35.90	1.4 × 10^6^	3.7 × 10^3^	0.72	1.11	1.10	59.13	336.34	1.1 × 10^5^
*DPPH*	1.05	0.99	−0.78	20.42	5.01	7.62	0.82	−0.21	−0.09	−0.67	0.65	0.42
*ABTS*	0.43	0.63	−0.16	39.21	4.16	6.44	0.47	1.98	5.52	−0.14	0.59	0.35
Flavonoids	222.93	14.35	−5.90	10.88	1957.05	222.42	0.67	−0.64	−0.77	−5.11	12.77	163.09
Phenols	9.6 × 10^3^	94.26	−19.67	18.59	95,261.54	942.48	0.40	0.41	1.42	−17.05	89.10	7.9 × 10^3^
Acidity	0.01	0.08	0.03	3.13	0.07	1.06	0.63	2.54	6.84	0.03	0.07	0.01
Betaine	1.8 × 10^5^	405.13	−1.43	5.08	1.8 × 10^6^	9.4 × 10^3^	0.73	0.12	−0.44	−1.24	390.39	1.5 × 10^5^

**Table 4:** Quality parameters of osmotically dehydrated beetroots, uncoated and coated with a CSoC-based biopolymer.

Sample	Acidity (% d.m.)	Phenols (mg eq.GA/100 g d.m.)	Flavonoids (mg ECA/100 g d.m.)	Betaine (mg/100 g d.m.)	*DPPH* IC_50_ (mg/mL)	*ABTS* IC_50_ (mg/mL)
OD_0_	1.30 ± 0.25 ^c^	1016.05 ± 21.50 ^fg^	238.80 ± 2.99 ^h^	3175.46 ± 21.36 ^de^	1.56 ± 0.13 ^c^	0.49 ± 0.01 ^bc^
ODC_0_	1.06 ± 0.07 ^abc^	1063.14 ± 24.73 ^g^	257.43 ± 0.32 ^i^	3343.13 ± 25.58 ^e^	1.37 ± 0.05 ^bc^	0.21 ± 0.00 ^a^
OD_7_	1.29 ± 0.12 ^bc^	678.02 ± 101.88 ^d^	127.87 ± 12.54 ^d^	3081.34 ± 73.88 ^d^	2.17 ± 0.01 ^e^	0.58 ± 0.06 ^d^
ODC_7_	0.99 ± 0.01 ^abc^	920.26 ± 23.50 ^ef^	221.73 ± 8.00 ^g^	3324.35 ± 18.89 ^e^	1.40 ± 0.01 ^bc^	0.20 ± 0.00 ^a^
OD_14_	0.85 ± 0.03 ^a^	468.28 ± 12.09 ^ab^	88.43 ± 3.65 ^b^	2365.16 ± 76.41 ^a^	1.89 ± 0.02 ^d^	0.70 ± 0.01 ^e^
ODC_14_	1.03 ± 0.07 ^abc^	682.43 ± 35.32 ^d^	172.34 ± 5.41 ^f^	2625.28 ± 91.13 ^b^	1.07 ± 0.05 ^a^	0.44 ± 0.00 ^bc^
OD_21_	0.98 ± 0.04 ^a^	468.06 ± 45.91 ^ab^	93.64 ± 6.15 ^bc^	2872.90 ± 61.83 ^c^	2.13 ± 0.17 ^e^	0.45 ± 0.04 ^bc^
ODC_21_	0.93 ± 0.08 ^a^	864.56 ± 15.50 ^e^	146.36 ± 3.24 ^f^	2666.85 ± 115.39 ^b^	1.21 ± 0.02 ^ab^	0.53 ± 0.00 ^cd^
OD_28_	0.85 ± 0.11 ^a^	458.09 ± 7.97 ^a^	63.37 ± 0.93 ^a^	2785.48 ± 63.7 ^bc^	2.22 ± 0.00 ^e^	0.51 ± 0.03 ^bc^
ODC_28_	1.09 ± 0.03 ^abc^	571.29 ± 14.24 ^cd^	105.38 ± 0.72 ^c^	3082.87 ± 56.24 ^d^	1.29 ± 0.03 ^b^	0.23 ± 0.01 ^a^

^a–i^ Means in the same column with different superscripts are statistically different (*p* ≤ 0.05). OD—osmotically dehydrated beetroots, ODC—osmotically dehydrated beetroots coated with CSoC biopolymer; acidity (% d.m.), phenols (mg eq.GA/100 g d.m.), flavonoids (mg ECA/100 g d.m.), betaine content (mg/100 g d.m.), DPPH (IC50 (mg/mL)), ABTS (IC50 (mg/mL)).

**Table 5:** Microbiological quality of osmotically dehydrated beets and osmotically dehydrated beets coated with *Camelina sativa* film stored at 4 °C after 7, 14, 21, and 28 days.

	Samples									
	Osmotically Dehydrated Beets					Osmotically Dehydrated Beets Coated with *Camelina sativa* Film				
	Storage Time (Days)									
Microorganisms:	0	7	14	21	28	0	7	14	21	28
*Escherichia coli* (CFU/g)	ND	ND	ND	ND	ND	ND	ND	ND	ND	ND
*Enterobacteriaceae* (CFU/g)	ND	ND	ND	ND	ND	ND	ND	ND	ND	ND
*Salmonella* spp. (CFU/g)	ND	ND	ND	ND	ND	ND	ND	ND	ND	ND
*Listeria monocytogenes* (CFU/g)	ND	ND	ND	ND	ND	ND	ND	ND	ND	ND
*Clostridium perfringens* (CFU/g)	ND	ND	ND	ND	ND	ND	ND	ND	ND	ND
Total number of microorganisms (CFU/g)	2600	1900	1200	1000	960	2400	1200	940	720	620
Total number of yeast and molds (CFU/g)	ND	ND	ND	ND	ND	ND	ND	ND	ND	ND

ND—not detected.

## Data Availability

The original contributions presented in the study are included in the article, further inquiries can be directed to the corresponding author.
